# Counterpoint: Flexibilization of Fasting for Laboratory Determination of the Lipid Profile in Brazil: Science or Convenience?

**DOI:** 10.5935/abc.20180192

**Published:** 2018-11

**Authors:** Maria Cristina de Oliveira Izar

**Affiliations:** Universidade Federal de São Paulo (UNIFESP), São Paulo, SP - Brazil

**Keywords:** Dyslipidemias, Cholesterol, Lipids, Triglycerides, Cholesterol, HDL, Cholesterol, LDL, Fasting

National and international guidelines for the management of dyslipidemias classically recommend measuring lipid profiles after fasting for at least 8 h.^1^^-^^3^ Lipid targets for assessing cardiovascular risk traditionally rely on plasma total-cholesterol and low-density lipoprotein-cholesterol (LDL-c) levels, with the latter being calculated by the Friedewald equation.^4^

Some imprecision due to low or high triglycerides in calculating LDL-cholesterol may affect cardiovascular risk assessment, the definition of a therapeutic target, and the need to intensify the treatment.^5^^,^^6^ Accurate results require triglyceride levels below 400 mg/dL, but above 100 mg/dL the calculated LDL-c starts to be underestimated, when compared to ultracentrifugation measurements. Another limitation to the use of the formula is that samples must not contain beta-VLDL, as in the case of type III hyperlipoproteinemia. When one of these conditions are not satisfied, the equation cannot be used due to imprecision.^5^^-^^7^

Other lipid parameters, such as apolipoprotein-B and non-high-density lipoprotein-cholesterol (non-HDL-C) reflect the pool of atherogenic lipoproteins and have emerged as good markers to improve cardiovascular risk assessment, and also to guide lipid-lowering therapy.^2^^,^^3^^,^^8^^,^^9^ These variables can be used in both the fasting and non-fasting states, and non-fasting lipoproteins are regarded as better atherosclerotic risk predictors, when compared with fasting ones, for they reflect remnant, atherogenic lipoproteins, with higher correlation with cardiovascular risk.^2^^,^^3^^,^^8^^,^^9^

To avoid the interference of triglycerides, direct measurements of LDL-cholesterol have been developed.^10^^,^^11^ but these techniques lack proper standardization, and were tested in few clinical trials that use LDL-c as target.^12^^,^^13^

Since then, many papers, as result of important and broad studies, were carried out comparing fasting and non-fasting lipid parameters, mainly total cholesterol, HDL-c, LDL-c and triglycerides, concluding that non-fasting lipids do not clinically differ from fasting ones, except for triglycerides, that require different reference values for non-fasting state.^14^^,^^15^

Here we present a second opinion for what has been stated in the article: *“Flexibilization of fasting for laboratory determination of the lipid profile in Brazil: science or convenience?”*

Our second opinion uses steps for building a scientific statement. The first step is to find an issue of interest to be debated. The second step requires full understanding of what is currently known about what is being explained. This basically deals with scientific publications, citations seeking other scientific papers, and books on the topic. Although it is possible to defer to the scientific consensus, you cannot really have a personal scientific viewpoint on anything without understanding what current research says about it.

Keep in mind that all scientific papers should be found in peer-reviewed well-reputed journals. It is best to approach scientific literature with no prior judgements; however, it can be a difficult task. After reviewing all relevant papers to the matter, it is possible to develop a scientific view and an opinion. If the scientific material collected reaches the same conclusion, it is unlikely that you can hold a different viewpoint at this moment. But, if some papers disagree, there is room for debate and to raise a plausible second opinion, if there is good research supporting this view. High-quality, well-designed studies, with a large number of participants, in the opposite direction of what had been stated, *do* reinforce the validity of a second opinion.

This article will address the interpretation, applications and limitations of a non-fasting lipid profile for daily clinical practice.

First, large observational data, with population-based studies and registries, including 111,048 women, 98,132 men, 12,744 children, and patients with diabetes, in which non-fasting lipid profiles were compared with those obtained under fasting conditions, have demonstrated that the maximal changes in plasma lipids and lipoproteins occurred between 1-6 hrs. after a usual meal. These trials have established that only minor changes occurred in response to habitual food intake in the majority of individuals.^14^^,^^16^^-^^19^ Total cholesterol, LDL-c, remnant cholesterol, varied 8 mg/dL, whereas HDL-c, apolipoprotein A1, apolipoprotein B, and lipoprotein(a) were not affected by fasting/non-fasting status. These data were derived from the Women’s Health Study, the Copenhagen General Population Study, the National Health and Nutrition Examination Survey, and the Calgary Laboratory Services in Canada.^14^^,^^16^^-^^19^

Among all studies, only minor increases in plasma triglycerides and minor decreases in total and LDL cholesterol concentrations were observed, in non-fasting conditions, with no change in HDL cholesterol concentrations. In subjects with diabetes, calculated LDL-c obtained 1-3 hours after a meal decreased 23 mg/dL, and could imply in statin withhold; however, when corrected for albumin, reflecting fluid intake, the difference disappeared, and was attributed to the fluid and *not* to the diet.^20^

Second, we live most of our time in non-fasting state. Non-fasting and fasting lipid concentrations vary similarly over time and are at least equivalent in the prediction of cardiovascular disease. In fact, data from the Calgary Laboratory Services in Canada demonstrated that in ~200,000 men and women, total cholesterol, HDL and LDL-cholesterol did not vary as a function of the period of fasting after the last meal.^17^

Third, reference plasma lipid, lipoprotein, and apolipoprotein concentration values based on desirable concentration cutoff points, do not vary when non-fasting, except for triglycerides, which should be flagged as abnormal in laboratory reports > 175 mg/dL. However, non-fasting triglycerides were better predictors than in the fasting state.^7^

Fourth, the risk of ischemic heart disease and myocardial infarction in 92,285 individuals from the Copenhagen General Population Study recruited from 2003 through 2014, could be predicted by non-fasting lipids (reported in Nordestgaard et al.^7^).

Fifth, a novel method to estimate LDL-C using an adjustable factor for the TG:VLDL-C ratio provided a more accurate guideline risk classification than the Friedewald equation.^21^ The authors used a large convenience sample of consecutive clinical lipid profiles obtained from 2009 through 2011 (n = 1,350,908), including children, adolescents, and adults in the United States). The sample was randomly assigned to derivation (n = 900,605) or validation (n = 450,303) data sets. Results closely matched those in the National Health and Nutrition Examination Survey (NHANES). This estimation method provided higher-fidelity estimates than the Friedewald equation. The greatest improvement in concordance occurred when classifying LDL-C lower than 70 mg/dL, especially in patients with high triglyceride levels. Indeed, there is a need for external validation, and assessment of its clinical importance. However, this novel method could be implemented in most laboratory reporting systems with virtually *no* cost.

Finally, what would be the problem to add convenience to science? Postprandial measurements are more practical and provide the patient a greater access to the laboratory, and also can decrease the number of missed working days and medical appointments due to missed tests; blood collection in the postprandial state is safer in several circumstances and help prevent hypoglycemia secondary to the use of insulin in patients with diabetes mellitus, in pregnant women, children, and elderly individuals, reducing complications and increasing adherence to the tests and to medical appointments; flexibilization of fasting for lipid profiling, can bring more comfort to the patient and greater amplitude of schedules in the laboratories, especially in the morning; technological advances in diagnostic methods, can mitigate the interference of sample turbidity when triglycerides are high.^22^

If, fasting is not routinely required for assessing the plasma lipid profile, some recommendations should be made in specific situations: 1) when non-fasting plasma triglyceride concentration exceed 440 mg/dL, consideration should be given to repeating the lipid profile in the fasting state; 2) laboratory reports should flag abnormal values based on desirable concentration cut-off points; 3) life-threatening or extremely high concentrations should trigger an immediate referral to a lipid clinic or to a physician with special interest in lipids.^7^^,^^22^
